# Knockout of the P2Y_6_ Receptor Prevents Peri-Infarct Neuronal Loss after Transient, Focal Ischemia in Mouse Brain

**DOI:** 10.3390/ijms23042304

**Published:** 2022-02-19

**Authors:** Stefan Milde, Guy C. Brown

**Affiliations:** Department of Biochemistry, University of Cambridge, Cambridge CB2 1QW, UK; stefanmilde@googlemail.com

**Keywords:** stroke, ischemia, microglia, phagocytosis, cell death, phagoptosis, neuronal death, delayed neuronal death, selective neuronal loss

## Abstract

After stroke, there is a delayed neuronal loss in brain areas surrounding the infarct, which may in part be mediated by microglial phagocytosis of stressed neurons. Microglial phagocytosis of stressed or damaged neurons can be mediated by UDP released from stressed neurons activating the P2Y_6_ receptor on microglia, inducing microglial phagocytosis of such neurons. We show evidence here from a small trial that the knockout of the P2Y_6_ receptor, required for microglial phagocytosis of neurons, prevents the delayed neuronal loss after transient, focal brain ischemia induced by endothelin-1 injection in mice. Wild-type mice had neuronal loss and neuronal nuclear material within microglia in peri-infarct areas. P2Y_6_ receptor knockout mice had no significant neuronal loss in peri-infarct brain areas seven days after brain ischemia. Thus, delayed neuronal loss after stroke may in part be mediated by microglial phagocytosis of stressed neurons, and the P2Y_6_ receptor is a potential treatment target to prevent peri-infarct neuronal loss.

## 1. Introduction

Stroke is one of the main causes of mortality and serious disability in the world [1]. Ischemic stroke is caused by blockage of an artery in the brain, resulting in rapid death of all cells within the area of lowest perfusion (the infarct), but also a delayed and selective neuronal loss in brain areas around the infarct (peri-infarct or penumbra areas) one to seven days after transient ischemia [2,3,4,5,6]. This delayed neuronal death after stroke is potentially preventable, so it is important to understand the mechanisms involved.

The mechanisms of delayed neuronal death after stroke are unclear [2,7], but one potential mechanism is microglial phagocytosis of live neurons, resulting in death of the engulfed neurons [8,9]. Microglial phagocytosis of live neurons and neuronal parts is known to occur during development, physiology, and pathology [10,11,12,13]. Microglial activation is associated with delayed neuronal loss in peri-infarct areas in rodent models of stroke [14,15,16,17]. We previously showed that transient brain ischemia induced by injection of endothelin-1 into the rodent brain caused a delayed loss of neurons, accompanied by microglial phagocytosis of neurons, and knock-out of the phagocytic receptor Mer tyrosine kinase (MerTK) or the opsonin MFG-E8 prevented both the delayed neuronal loss and long-term functional deficits [18]. This suggested that microglial phagocytosis contributed to the delayed neuronal loss after stroke. However, MerTK and MFG-E8 can mediate phagocytosis of dead cells and debris, which may be beneficial after stroke [19,20,21], so we were interested in other receptors regulating microglial phagocytosis of neurons as potential treatment targets.

The P2Y_6_ receptor (P2Y_6_R) is expressed on microglia and mediates microglial phagocytosis when activated by extracellular uridine diphosphate (UDP) released by stressed neurons [22,23]. We have previously shown that addition of UDP to neuronal-glial co-cultures results in microglial phagocytosis of live neurons, and that a P2Y_6_R inhibitor (MRS2578) prevents neuronal loss induced by UDP, lipopolysaccharide (LPS) and amyloid beta in culture, and prevents neuronal loss induced by injection of LPS into rat striatum [24]. More recently, we showed that chronic, peripheral LPS induced neuronal loss in the brains of mice and this neuronal loss was prevented by knockout of P2Y_6_R [25]. We also found that injection of amyloid beta into the brains of mice induced microglial phagocytosis of neurons, loss of neurons, and memory deficits, all of which were prevented in P2Y_6_R knockout mice [26]. Similarly, we used a chronic model of neurodegeneration (i.e., mice expressing P301S TAU) and found that crossing these mice with P2Y_6_R knockout mice prevented the brain neuronal loss and memory deficits [26]. Thus, we were interested to know whether P2Y_6_R knockout could prevent the loss of peri-infarct neurons after transient brain ischemia. To do this, we induced transient brain ischemia by injection of endothelin-1 into mouse brains, as this induces relatively mild damage and small infarcts [18,27,28] which may be more likely to benefit from inhibition of microglial phagocytosis. 

## 2. Results

In order to test whether blocking microglial phagocytosis can prevent neuronal loss in peri-infarct areas after stroke, we conducted a small study in P2Y_6_ receptor (P2Y_6_R) knockout and wild-type mice. We induced focal ischemia in a mouse brain by injecting the vasoconstrictor endothelin-1, which induces transient ischemia, followed by delayed neuronal loss [27,28]. 

**Figure 1:**
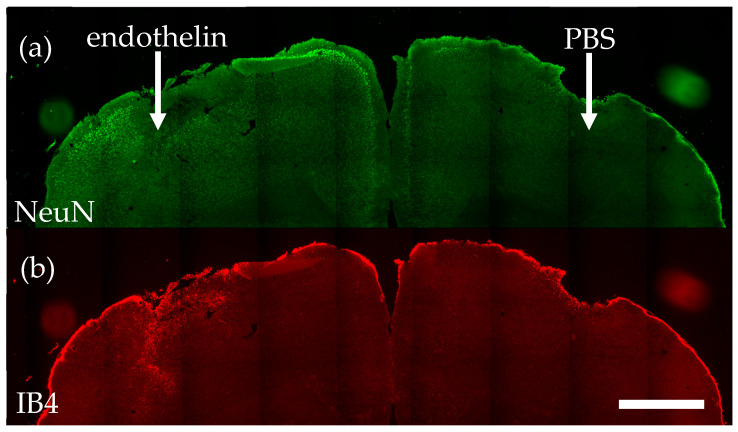
Endothelin-1 injection into wild-type mouse cortex induces neuronal loss and microglial activation, measured seven days later. (**a**) Representative coronal brain section stained using antibodies to NeuN, revealing neuronal nuclei. (**b**) Same section stained using IB4, revealing activated microglia. Scale bar = 1 mm.

We injected endothelin-1 into the right sensorimotor cortex (and vehicle PBS into the left motor cortex) of four wild-type and four P2Y_6_R knockout mice, and seven days later killed the mice. We then sectioned and stained the brains. The endothelin injection induced a small infarct area with zero neurons (visualized with antibodies to NeuN, an antigen specific to neuronal nuclei, Figure 1a) and microglial activation (visualized with isolectin B4 (IB4), which specifically binds activated microglia, Figure 1b). The injection of vehicle control (PBS) resulting in physical damage only, without microglial activation or neuronal loss (Figure 1a,b). 

**Figure 2:**
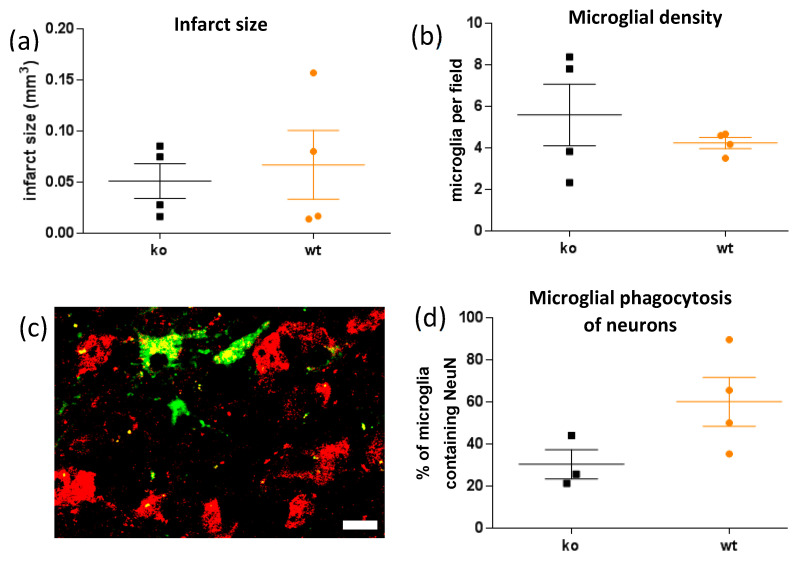
P2Y_6_R knockout (ko) has no effect on infarct size or microglial density relative to wild-type mice (wt), but reduces neuronal nuclear material within microglia. (**a**) Endothelin-1-induced infarct size, defined as area lacking neuronal nuclei. T-test of significance of difference yields t = 0.42, *p* = 0.69. (**b**) Microglia per field in peri-infarct area. t = 0.90, *p* = 0.40. (**c**) Confocal image of NeuN and IB4 stained peri-infarct area showing NeuN+ neuronal nuclear material (red) within microglia (green). Scale bar = 10 microns. (**d**) Proportion of microglia in the peri-infarct area with NeuN+ material inside. t = 2.00, *p* = 0.10. Mean and SEM error bars are shown. Each data point is the mean for a mouse.

The infarct size was similar in wild-type and P2Y_6_R knockout mice (Figure 2a). The density of microglia within the peri-infarct area was similar in wild-type and P2Y_6_R knockout mice (Figure 2b). Peri-infarct microglia contained NeuN+ neuronal debris within the microglia (Figure 2c), indicating microglial phagocytosis of neuronal nuclei. This phagocytosis was reduced by half in P2Y_6_R knockout mice (Figure 2d), although the difference was not significant (*p* = 0.10), suggesting less phagocytosis of neurons in the knockout.

**Figure 3:**
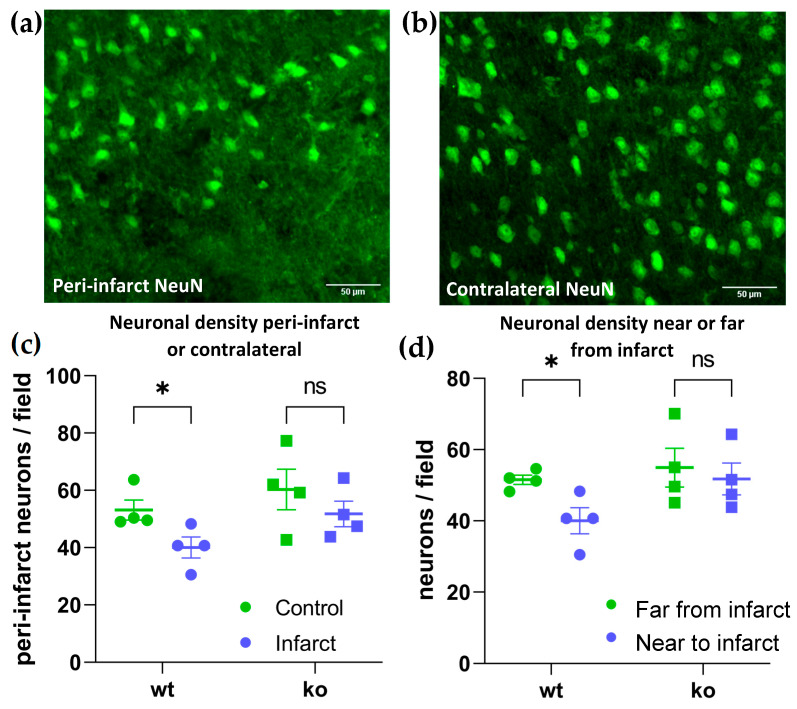
Knockout of the P2Y_6_ receptor prevents peri-infarct neuronal loss after transient, focal ischemia induced by endothelin-1 injection into mouse brain. (**a**) Representative peri-infarct brain section stained for NeuN, revealing neuronal nuclei in wild-type cortex injected with endothelin-1. Scale bars = 50 microns. (**b**) Representative contralateral section stained for NeuN in wild-type cortex injected with PBS. (**c**) Neurons per field in peri-infarct area on endothelin-1 (infarct) or PBS injected (control) side of motor cortex in wild-type or P2Y_6_R knockout mice. * *p* = 0.029, ns = 0.133. (**d**) Neurons per field in peri-infarct area (0–200 microns from infarct) and further from infarct (200–1000 microns from infarct) on the endothelin-1 side of motor cortex in wild-type or P2Y_6_R knockout mice. * *p* = 0.013, ns = 0.509. Data were analyzed by two-way ANOVA and Sidak’s multiple comparison’s test.

In wild-type mice, the infarct was surrounded by an area (from 0 to 200 microns from the infarct boundary) with reduced neuronal density (Figure 3a,b). Quantification of the neuronal density within this peri-infarct area indicated there was a significant loss of neurons in this area measured relative to either the adjacent area further from the infarct (200–1000 microns from the infarct boundary) or the anatomically-matched area on the contralateral hemisphere (control) in wild-type mice (Figure 3c,d). However, this peri-infarct neuronal loss was prevented in P2Y_6_R knockout mice. In other words, P2Y_6_R knockout mice had no significant neuronal loss in the peri-infarct area (Figure 3c,d). Thus, we conclude that P2Y_6_R knockout prevents peri-infarct neuronal loss.

## 3. Discussion

We found that knockout of P2Y_6_R in mice prevented neuronal loss in peri-infarct brain areas after transient, focal ischemia. These findings are consistent with this neuronal loss being due to microglial phagocytosis of stressed neurons. Kainate-stressed neurons have previously been shown to release UDP that activates P2Y_6_R on microglia to induce microglial phagocytosis of these stressed neurons [22]. Thus, it is possible that stressed neurons in peri-infarct areas after stroke may release UDP activating microglial phagocytosis of such neurons. 

We have previously shown that P2Y_6_R knockout prevents neuronal loss, memory loss, and microglial phagocytosis of neurons induced by injection of amyloid beta into the mouse brain [26], reduces neuronal loss and memory loss when crossed with the P301S TAU model of tauopathy [26], and prevents dopaminergic neuronal loss in the substantia nigra induced by peripheral endotoxin [25]. Similarly, injection of the P2Y_6_R antagonist MRS2578 into the brain prevented LPS/endotoxin-induced neuronal loss [24], and others have found that injection of the P2Y_6_R antagonist MRS2578 into the brain prevented 6-hydroxydopamine-induced loss of dopaminergic neuronal loss in the substantia nigra [29]. P2Y_6_R has been found to promote cytokine and chemokine release from monocytes, and thus has been suggested to be pro-inflammatory in microglia [23,30]. However, inhibition or knockout of P2Y_6_R has no apparent effect on the inflammatory response of microglia [24,26,31]. On the other hand, P2Y_6_R may regulate functions other than microglial phagocytosis. For example, P2Y_6_R has been shown to regulate differentiation of NK (natural killer) cells [32]. However, this has no obvious relevance to stoke damage in the brain.

Microglial phagocytosis has been shown to contribute to delayed neuronal loss after stroke using diverse means of blocking phagocytosis. For example, we found that that knockout of MFG-E8 or MerTK could reduce delayed neuronal loss after ischemic stroke in mice and rats respectively [18]. Others showed that knockdown of TMEM16F, which mediates reversible phosphatidylserine exposure on neurons after ischemia, prevented microglial phagocytosis of stressed neurons and reduced motor deficits after transient ischemic stroke in rats [33]. Activation of complement component C3 produces potent opsonins to tag neurons for phagocytosis, and an inhibitor of C3 activation, Crry, prevented phagocytosis of stressed-but-salvageable neurons in peri-infarct areas, and reduced functional deficits in a mouse model of stroke [34,35]. Thus, there is evidence from a variety of sources that microglial phagocytosis may contribute to neuronal loss after stroke (as reviewed in [9]).

Our findings suggest the possibility that a P2Y_6_R inhibitor might be used to prevent peri-infarct neuronal loss by blocking microglial phagocytosis of the neurons. A P2Y_6_R inhibitor (MRS2578, peripherally given i.p.) has previously been reported to increase brain atrophy and functional deficits after transient MCAO [31]. However, the specificity of MRS2578 and its ability to cross the BBB are unknown, and peripheral MRS2578 can cause hypotension [36], which could exacerbate ischemic brain damage. To elucidate these different experimental findings, it would be useful to test: (i) whether P2Y_6_R knockout reduces functional deficits against transient middle cerebral artery occlusion, and (ii) whether MRS2578 given i.p. enters the brain, blocks microglial phagocytosis, and/or has off-target effects.

Endothelin-1 injection induces relatively small infarcts, similar to lacunar strokes (i.e., strokes induced by blockage of small blood vessels [27,28]), and it is possible that blocking microglial phagocytosis is more beneficial in such small strokes since there is less damage and debris to be removed. However, we did not test this. It would be important to test whether P2Y_6_R knockout or inhibition is beneficial in stroke models with larger infarcts. However, this study encourages further research to determine whether inhibition of the P2Y_6_ receptor is beneficial after stroke by preventing delayed neuronal loss by microglial phagocytosis.

## 4. Materials and Methods

P2ry6 knockout (P2ry6^−/−^) mice were kindly provided by Bernard Robaye (ULB Brussels) and these were bred with C57BL/6 mice (Charles River Laboratories) for at least six generation as P2ry6^+/−^ mice. These mice were used to establish homozygous WT and P2ry6^−/−^ sub-lines. In offspring from these sub-lines, littermates were randomly assigned to control and endothelin-1 treatment groups. Four wild-type (two male and two female) and four P2ry6^−/−^ mice (two male and two female) were used, all between four and six months of age.

Induction of ischemia and subsequent analysis of the brains was as described in [18]. Endothelin-1 (Bachem, 0.75 μg in 1 μL of PBS) was injected (at 6 μL/h) into the right sensorimotor cortex, and the same volume (1 μL) of PBS (phosphate buffered saline) was injected into the left sensorimotor cortex, of adult (4–5-month-old) wildtype or P2ry6^−/−^ mice using a 26-gauge needle on a stereotaxic frame (Kopf Instruments) under isoflurane anesthesia. Injection coordinates were antero-posterior (AP) + 0.6 mm, medio-lateral (ML) ± 2.2 mm, dorso-ventral (DV) −1.7 mm from Bregma, flat skull. Mice were allowed to recover, and tissues collected seven days after injection.

Mice were given terminal anaesthesia (150 µL Euthatal (200 mg pentobarbital per ml) intraperitoneal (i.p.)) and perfused transcardially, through a 25-gauge needle, with 20 mL PBS pH 7.4 followed by 60 mL 4% paraformaldehyde (PFA), pH 7.4 using a perfusion pump with flow rate of 4 mL/min. Following perfusion, brains were removed and post-fixed overnight in 4% PFA, pH 7.4 for 16 h at 4 °C. Brains were then washed three times in 1× PBS and stored in 30% sucrose solution until sectioning. Brain sections were cut to 20 µm thickness using a Compresstome VF-200 vibratome (Precisionary Instruments, Natick, MA, USA), collected on Superfrost Plus slides (Thermo Fisher; Waltham, MA, USA) and dried overnight. Serial coronal sections (25 µm) through the whole brain were collected using a sliding microtome and placed in PBS as free-floating sections.

Immunostaining of brain slices was carried out at room temperature unless indicated otherwise. Brain slices were re-hydrated for 1 h in PBS and heat-mediated antigen retrieval was carried out at 95 °C for 20 min in citrate buffer (10 mM sodium citrate, 0.05% Tween 20, pH 6.0). Following washes in PBS (6 × 10 min), slices were permeabilized in PBS with 0.5% Triton X-100 for 10 min followed by 1 h incubation in blocking solution (50% normal goat serum in PBS). Slices were then incubated with Anti-NeuN (Millipore, Burlington, MA, USA, mouse monoclonal, 1:500) and biotinylated isolectin-B4 (Sigma, St. Louis, MO, USA, 1:200) at 4 °C overnight. Sections were washed, incubated with donkey anti-mouse-Cy3 antibody (Jackson ImmunoResearch, Ely, U.K., 1:100), washed, and treated with Streptavidin-Alexa Fluor 647 (Invitrogen, Waltham, MA, USA, 1:500), 2 h each, at RT. Imaging was carried out on an Olympus FV1000 upright laser-scanning confocal microscope with a 60×, 1.35NA oil immersion objective using 488, 559 and 635 nm laser lines. 

All image analysis was carried out using ImageJ 1.49 software and all manual counting and quantification was performed blinded to genotype and treatment condition. Four brain sections were analyzed per animal, with both right and left sides of the sensorimotor cortex included in the analysis. Using anti-NeuN and IB4-stained sections, the infarct was defined as the cortical area lacking NeuN+ neurons, and the infarct volume was calculated from contiguous sections. Neuronal density in the peri-infarct area was quantified as NeuN+ cells counted manually in the area between 0 and 200 microns from the infarct boundary. Control neuronal density was quantified as NeuN+ cells in the area between 200 and 1000 microns from the infarct boundary, or alternatively as NeuN+ cells in anatomically matched areas of the other cerebral hemisphere (injected with PBS). Three fields were counted on each side per animal. Microglial density in the peri-infarct area was manually counted as IB4+ cells in the same areas defined for counting NeuN+ cells. Microglial phagocytosis of neuronal material was quantified as NeuN+ material within IB4+ cells.

Statistical analysis was carried out using GraphPad Prism 9 and the statistical tests are indicated in the figure legends.

## Data Availability

The data presented in this study are available on request from the corresponding author.
